# Thyroid hormones and frailty in older adults: systematic review and dose–response meta-analysis

**DOI:** 10.1186/s12877-025-05748-5

**Published:** 2025-02-17

**Authors:** Wen-Chun Chia, Yang-Ching Chen, Shuang-ling Xiu, Sen-Te Wang

**Affiliations:** 1 Department of Family Medicine, Taipei Medical University Hospital, Taipei, Taiwan; 2 Department of Family Medicine, Wan Fang Hospital, Taipei Medical University, Taipei, 116 Taiwan; 3 Department of Family Medicine, School of Medicine, College of Medicine, Taipei Medical University, Taipei, Taiwan; 4 Department of Endocrinology, Xuanwu Hospital, Capital Medical University, Beijing, China; 5 Present Address: Lifestyle and Healthcare Management Center, Joyce Clinic, Taipei, Taiwan; 6 Present Address: Department of Family Medicine, Union Clinic, Taipei, Taiwan; 7 Taipei Medical University Hospital, No. 252, Wuxing St., Xinyi Dist., Taipei City, 110 Taiwan (R.O.C.)

**Keywords:** Frailty, TSH, Thyroid hormone

## Abstract

**Objective:**

To investigate (1) whether the association of thyroid hormone with frailty risk is linear or nonlinear and (2) which range of thyroid hormones or thyroid stimulating hormone (TSH) is more associated with a higher risk of frailty in older adults.

**Design:**

Systematic review and dose–response meta-analysis.

**Methods:**

Medical electronic databases were searched for cross-sectional or longitudinal studies, published from database inception to February 2022. We focused on the relationship between TSH and frailty. Data on TSH reference range, TSH exposure categories, sample size of each exposure category, and adjusted odds ratios (ORs) for frailty with 95% confidence interval (CI) were extracted. In the dose–response meta-analysis, we set the OR for frailty as 1 at 0.3 mIU/L TSH.

**Results:**

The systematic review included 10 studies, whereas the meta-analysis included 3 studies (*n* = 6388). TSH levels ranged from 0.3 to 4.8 mIU/L, and the dose–response meta-analysis revealed a significant J-shaped association (*p* = 0.0071). Frailty OR (95% CI) increased from 1.30 (1.06–1.59) for 2.7 mIU/L TSH to 2.06 (1.18–3.57) for 4.8 mIU/L TSH.

**Conclusions:**

A significant nonlinear, J-shaped association was noted between TSH level and frailty. TSH levels within the upper half (2.7–4.8mIU/L) of reference range was noted to significantly higher risk of frailty; by contrast, those in the lower half (0.6–1.5 mIU/L) had a lower risk of frailty, though not significantly so.

**Trail registration:**

This systematic review was registered with PROSPERO (registration number: CRD42022299214).

**Supplementary Information:**

The online version contains supplementary material available at 10.1186/s12877-025-05748-5.

## Introduction

Frailty is characterized by loss of biological reserves across multiple organ systems, failure of homeostatic mechanisms, and vulnerability to physiological decompensation after minor stressor events [1]. In people aged > 65 and > 85 years, frailty prevalence is approximately 10% and 25%–50%, respectively [2]. The major frailty models are the phenotype model [3] and the cumulative deficit model [4]. The frailty phenotype includes five domains: unintentional weight loss, self-reported exhaustion, low energy expenditure, slow gait speed, and weak grip strength [3]. The frailty status is categorized by the number of domains: robustness (no domain), pre-frailty (one or two domains), and frailty (three or more domains). The frailty index is defined by baseline variables such as signs and symptoms, abnormal laboratory profiles, disease states, and disabilities; it is simply calculated based on the presence or absence of each variable as a proportion of the total, scored from 0 to 1 [4]. The two aforementioned models, although different, can be considered complementary. The frailty phenotype, a categorical outcome, is based on clinical signs and symptoms and can be applied at the first contact with the affected individuals. Although the frailty index is available only after a comprehensive clinical assessment, its’ continuous trait makes it more predictive in severity and more sensitive to intervention or follow-up [5].

The endocrine system is considered a key system involved in frailty through complex interrelationships with the brain, immune system, and skeletal muscles. Thyroid hormone signaling is required for skeletal muscle contractile function, metabolic processes, myogenesis, and regeneration [6]. Thyroid hormone levels change with age: thyroid stimulating hormone (TSH) levels increase, free triiodothyronine (fT3) levels decrease, and free thyroxine (fT4) levels remain stable [2]. Changes in TSH and fT3 levels with aging are associated with alterations in signaling pathways and nutritional status, and the resulting cumulative illness may be linked to alterations in muscle metabolism and structure [7]. Loss of muscle mass and strength with aging, defined as sarcopenia, leads to physical function loss. The aforementioned conditions are consistent with the definition of frailty [2].

To the best of our knowledge, no systematic review nor any meta-analysis has discussed the association between thyroid hormones and frailty. Most of the included cross-sectional studies only modeled linear relationships, and these linear models disagreed with regard to the direction of correlation; only one study [8] demonstrated a J-shaped association of thyroid hormone with frailty. Considering the close relationship between sarcopenia, a key component of frailty, and thyroid hormones, Szlejf et al. reported a cross-sectional study involving 6974 participants [7]. Their results revealed that subclinical thyroid dysfunction is not associated with sarcopenia and its defining components; however, in older adults, TSH ranged from 0.4 to 4 mIU/mL exhibited a U-shaped association with sarcopenia and low muscle strength. This study focuses on the broader concept of frailty, recognizing that while sarcopenia plays a significant role, frailty encompasses a wider range of health deficits.

Therefore, understanding the association between thyroid hormone levels and the risk of frailty in older individuals is essential. This study aims to investigate (1) whether the association of thyroid hormone level with frailty risk is linear or nonlinear and (2) which range of thyroid stimulating hormone (TSH) levels is more associated with a higher risk of frailty in older adults.

## Methods

### Study selection and search method

This study was conducted according to the Preferred Reporting Items for Systematic Reviews and Meta-Analyses standards [9]. Two authors WCC and STW conducted an electronic database search of PubMed, Embase, Web of Science, EBSCOhost, and the Cochrane Library for English-language articles on both thyroid hormone and frailty published from database inception to February 2022. Observational studies and which study population aged over 50 were included in this study. During full-text assessment, we included cross-sectional and longitudinal studies in qualitative study. However, none of the longitudinal studies met the data requirements for the meta-analysis. We excluded animal studies, studies discussing only thyroid autoantibodies, review studies, and non-English studies. Any disagreements between the two aforementioned authors were resolved through discussion with a third author (YCC).

**Fig. 1:**
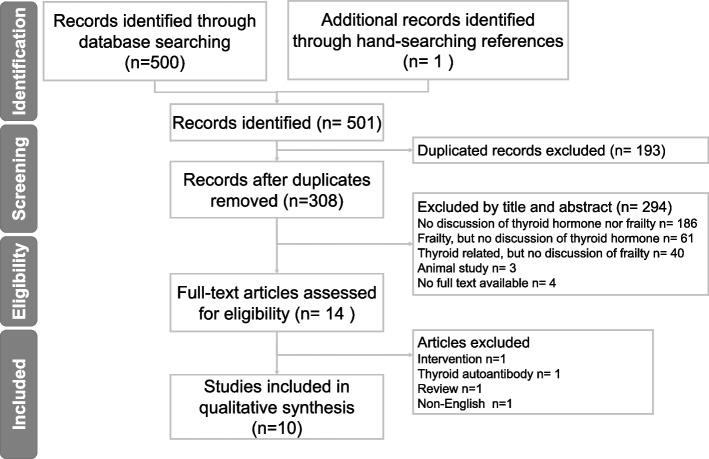
Flow diagram showing preferred reported items in systematic reviews and meta-analyses

To address this question, we formulated our research question using the PICO format and its MeSH term: Population (older adults), Intervention (thyroid hormone levels), Comparison (different levels of thyroid hormones or TSH), and Outcome (frailty). Figure 1 and Supplementary Table 1 presents the study selection process and the detailed search strategy, respectively.

### Data extraction and assessment of risk of bias

First author, publication year, study design, country, total number of participants, mean participant age and standard deviation, thyroid hormone outcome details, frailty definition, adjusted confounding factors and the main result were extracted from the included studies by two authors WCC and STW. We also emailed the author of included article for retrieving data. The quality of each study was assessed using Quality Assessment Tool for Observational Cohort and Cross-Sectional Studies from National Heart, Lung, and Blood Institute (NHLBI) [10]. Publication bias across individual studies was assessed by Egger’s test.

### Statistical analysis

For the meta-analysis, we focused on the relationship between TSH and the odds ratio (OR) for frailty, which was defined based on the frailty phenotype. For quantitative analysis, we extracted reference ranges of TSH levels, TSH exposure categories, sample sizes of the individual exposure categories, adjusted ORs for frailty with their 95% confidence interval (CI), and confounding factors for multivariable analysis. If the total number and the number of cases by TSH exposure categories could not be extracted directly, we estimated them based on the total number of included cases and the prevalence of frailty. The calculations are detailed in Supplementary Table 2. To reduce the influence of the confounding factors, the effect sizes were extracted from the adjusted ORs. We only extracted the data at the first assessment, rather than at the follow-up assessment.

The ORs (95% Cis) were pooled using the random-effects model. Statistical heterogeneity among studies was assessed using the Q and *I*^2^ tests, and an *I*^2^ of > 50% was considered to indicate considerable heterogeneity based on Cochrane handbook [11]. The weighting of each study was calculated using the standard error of log-transformed ORs with the inverse variance method. We standardized the varying TSH exposure categorizations across different studies by dividing TSH levels into low, intermediate, and high groups. The “intermediate group” refers to the group with TSH levels falling between the low and high TSH groups. Because of the possibility of a J-shaped association, we defined the reference group as the intermediate group to investigate the association of high or low versus intermediate TSH levels and frailty risk (Supplementary Table 2). We also performed a subgroup analysis for sex.

A dose–response meta-analysis was then performed by using the generalized least squares trend estimation, developed by Greenland and Longnecker [12]. To evaluate the associations between TSH levels and frailty risk, we used the restricted cubic spline (RCS) model, as described by Orsini et al. [12]. Following their recommendations, we placed four knots at the 5th, 35th, 65th, and 95th percentiles of the aggregated TSH exposure distribution [13], to evaluate the associations between TSH levels and frailty risk. The level in each TSH exposure category was extracted from the articles for calculating the midpoint of each exposure category (Supplementary Table 2). We observed inconsistent cutoff points of TSH exposure among the included studies, such as Veronese et al. [14] reported more precise categories in TSH levels < 1.1 mIU/L. Hence, we performed a subgroup dose–response meta-analysis.

All statistical analyses were performed using R (version 4.1.2’ R Foundation for Statistical Computing, Vienna, Austria).

## Results

In total, 501 articles were obtained in the initial search. After the eligibility assessment, only 14 studies remained. Among them, one interventional study [15], one study only discussing thyroid autoantibodies [16], one review study [17], and one non-English study [18] were excluded. Finally, 10 studies were included in further analysis (Fig. 1).

### Study characteristics

The characteristics of the 10 included studies, including 5 cross-sectional and 5 cohort studies, are summarized in Table 1. Their sample sizes ranged from 112 to 3943. Regarding thyroid hormone outcome data, nine studies collected thyroid hormone level data directly, whereas one [19] classified thyroid hormone levels based on whether subclinical hypothyroidism, hyperthyroidism, or euthyroidism. Regarding frailty data, four studies reported continuous data (i.e., frailty index or score), whereas the remaining six studies reported categorical data; among the six studies, five reported the frailty phenotype.

**Table 1:** Study characteristics of the 10 included studies

First author, Year	Country	Study Design	Total participants / setting	Age (mean ± SD) years	Thyroid hormone	Frailty definition	Adjusted confounding factors	Main results
Yeap [20], 2012	Australia	Cross-sectional study	3943 men/ Community	75.2 ± 4.1	TSH(0.4–4.0mIU/L), FT4(10–23 pmol/L)	Frailty phenotype criteria: Frail Nonfrail	Age, BMI, smoke status, diabetes, social support, impairment of seeing or hearing, testosterone and Insulin–like growth factor–I level	Nonsignificant association noted between TSH and frailty (*p* > 0.5). FT4 had the highest odds for frailty in two quartiles (Q3:Q1, OR = 1.32, 95% CI = 1.01–1.73; Q4:Q1, OR = 1.36, 95% CI = 1.04–1.79; *p* = 0.010). Neither subclinical hyperthyroidism (OR = 0.69, 95% CI = 0.21–2.31) nor subclinical hypothyroidism (OR = 1.17, 95% CI = 0.90–1.53) was significantly associated with frailty
Virgini [19], 2015	Switzerland	Prospective cohort study	1455 men/ Community	73.6 ± 5.8	Subclinical hyperthyroidism, subclinical hypothyroidism, euthyroidism TSH(0.55–4.78mIU/L), FT4( 0.8–1.75 ng/dL)	Frailty phenotype criteria: Robust group Prefrail and frail group	age, race, BMI and clinicalcenter	Compared with those with euthyroid, men with subclinical hyperthyroidism had an increased likelihood of high frailty status (adjusted OR = 2.48, 95% CI = 1.15–5.34)
Bertoli [21], 2017	Italy	Observational study	112/ 62 hospitalized, 50 outpatient	79.1 ± 7.0	TSH(0.35–4.5μIU/mL), FT4(0.8–1.75ng/dL), FT3(2.3–4.2 pg/mL)	Frailty score	NA	Frailty score was significantly correlated with FT3 (*p* < 0.0001), but not FT4 (*p* = 0.1974)
Veronese [14], 2017	Italy	2571 cross-sectional, 1732 longitudinal	3099 (1245 men, 1854 women)/ community	Men 73.2 ± 6.5 Women 74.7 ± 7.3	TSH (0.3 and 4.2 mUI/L) Quintiles cutoffs for men: 0.7, 1.0, 1.3, and 2, while for women 0.8, 1.1, 1.5, and 2.5 mUI/L	Frailty phenotype criteria: Frail Nonfrail	Age, BMI, smoke status, alcohol drinker, education, monthly income, ADL, geriatric depression, MMSE scores, Charlson comorbidity score, eGFR, number of drugs	With the third quintile of serum TSH (Q3) as the reference group, the highest quintile (Q5) was associated with the highest frailty risk in men (OR = 1.55, 95% CI = 1.03–2.33) and in women (OR = 1.97, 95% CI = 1.59–2.45)
Bano [8], 2018	Netherlands	Prospective cohort study	9,640/ NA	64.9 ± 9.7	TSH (0.40 to 4.0 mIU/L), FT4( 0.86 to 1.94 ng/dL)	Frailty index: 45-item	age, sex, cohort, smoking, alcohol, and education	TSH (*p* < 0.0003) and FT4 (*p* < 0.0001) with frailty at baseline
Pasqualetti [22], 2018	Italy	Longitudinal study	619/ hospitalized	83.8 ± 7.4	TSH(0.4–4.0mIU/L), FT4(0.70–1.70ng/dL), FT3( 2.7–5.0 pg/mL)	MPI score: > 0.66 = frailty; 0.34–0.66 = Prefrail 0.34 = Robust	age, sex, MPI, FT3, LDH, Hb, CRP and albumin	MPI score was inversely and strongly correlated with FT3 (*p* < 0.001) and moderately and positively correlated with FT4 (*p* < 0.05)
Arosio [23], 2020	Italy	Cohort study	593/community or nursing home	80.1 ± 15.7	TSH(0.27–4.2µIU/ml), FT4(0.9–1.7ng/dl), FT3(2.3–4.4 pg/ml)	Frailty index: 30 items	sex, age and study center	Correlation of frailty index with FT3 (*ρ* = − 0.281, p < 0.001), TSH (*ρ* = − 0.223, *p* = 0.003) was negative Correlation of frailty index with FT4 was positive (*ρ* = 0.189, *p* = 0.001)
Xiu [24], 2020	China	Cross-sectional study	240 (T2DM)/ NA	68.9 ± 6.9	TSH(0.55–4.78mIU/mL), FT4(0.89–1.76ng/dL), FT3(2.3–4.2 pg/mL)	Frailty phenotype criteria: Frail Prefrail Robust	age, sex, 25(OH) D3, eGFR, FT3	Logistic regression showed that low FT3 was significantly associated with an increased risk of frailty (OR = 4.53, 95% CI = 1.89–10.83; *P* = 0.001)
Bhalla [25], 2021	USA	Cross-sectional study	150/ inpatients	70.0 ± 6.2	TSH(0.5-5µIU/ml), FT4(0.70–1.48ng/dL), FT3(1.50–4.20 pg/ml)	Frailty index: 30 items	age	Patients with lower TSH (0.31 ± 0.11 µIU/mL) had higher mean frailty index (0.25 ± 0.12), and patients with normal TSH (1.84 ± 0.84 µIU/mL) had lower mean frailty index (0.15 ± 0.07; *p* < 0.001) An association of FT3 levels with FI was inverse (*p* = 0.13), and it disappeared when age was adjusted for (*p* = 0.4)
Liu [26], 2021	China	Cross-sectional study	146/ inpatients	85.0 ± 8.2	TSH(0.35–4.94mIU/L), FT4(9.01–19.05pmol/L), FT3(2.63–5.70pmol/L), T4(62.88–150.80 nmol/L), T3(0.88–2.44 nmol/L)	Frailty phenotype criteria: Frail Prefrail Robust	age, sex, BMI, smoking, and HbA1c	Frailty was significantly associated with serum TSH (OR = 1.258) and T3 (OR = 0.102) levels

*TSH* thyroid stimulating hormone, *FT4* free thyroxine, *FT3* free triiodothyronine, *TPOAb* thyroperoxidase autoantibody, *TgAb* thyroglobulin autoantibody, *MPI* multi prognostic index, *T2DM* type 2 diabetes mellitus, *OR* odds ratio, *CI* confidence interval, *SD* standard deviation, *BMI* body mass index, *HbA1c* glycosylated hemoglobin, *MPI* multi-prognostic Index, *LDH* lactic dehydrogenase, *Hb* haemoglobin, *CRP* C-reactive protein, *ADL* Activities of Daily Living, *MMSE* Mini–Mental State Examination, *eGFR* estimated Glomerular filtration rate

### Quality assessment

Table 2 presents data on the methodological quality of the included studies according to the NHLBI’s quality assessment tool. The rating for questions 1–4, which address the study objective, population, participation rate, and prespecified inclusion and exclusion criteria, is generally “yes”. The rating for questions 5–7, which adress sample size justification, exposure prior to outcome measurement, and association of exposure and the outcome, is generally “no, due to the included studies are mostly cross-sectional. Among questions 8,9,11, the exposure and outcome measure of all included studies can vary in level and with clearly defined measurement. Among questions 10, 12, 13, exposure assessment time, outcome assessor blinded to exposure and loss follow up rate was mostly non-available in cross sectional included studies. Among questions 14, 9 out of 10 included studies adjusted confounding factors. The Egger’s regression test yielded a non-significant result (*p* = 0.731422), suggesting no evidence of publication bias.

**Table 2:** Summary of risks of bias of the 10 included studies

	Yeap [20], 2012	Virgini [19], 2015	Bertoli [21], 2017	Veronese [14], 2017	Bano [8], 2018	Pasqualetti [22], 2018	Arosio [23], 2020	Xiu [24], 2020	Bhalla [25], 2021	Liu [26], 2021
1. Was the research question or objective in this paper clearly stated?	Y	Y	Y	Y	Y	Y	Y	Y	Y	Y
2. Was the study population clearly specified and defined?	Y	Y	Y	Y	Y	Y	Y	Y	Y	Y
3. Was the participation rate of eligible persons at least 50%?	Y	Y	Y	Y	Y	Y	Y	Y	Y	Y
4. Were all the participants selected or recruited from the same or similar populations (including the same time period)? Were inclusion and exclusion criteria for being in the study prespecified and applied uniformly to all participants?	Y	Y	N	Y	Y	Y	Y	Y	Y	Y
5. Was a sample size justification, power description, or variance and effect estimates provided?	N	N	N	N	N	N	N	N	N	N
6. For the analyses in this paper, were the exposure(s) of interest measured prior to the outcome(s) being measured?	N	Y	N	N	N	N	N	N	N	N
7. Was the timeframe sufficient so that one could reasonably expect to see an association between exposure and outcome if it existed?	N	Y	N	Y	N	N	N	N	N	N
8. For exposures that can vary in amount or level, did the study examine different levels of the exposure as related to the outcome (e.g., categories of exposure, or exposure measured as continuous variable)?	Y	Y	Y	Y	Y	Y	Y	Y	Y	Y
9. Were the exposure measures (independent variables) clearly defined, valid, reliable, and implemented consistently across all study participants?	Y	Y	Y	Y	Y	Y	Y	Y	Y	Y
10. Was the exposure(s) assessed more than once over time?	NA	Y	NA	N	Y	N	N	N	N	N
11. Were the outcome measures (dependent variables) clearly defined, valid, reliable, and implemented consistently across all study participants?	Y	Y	Y	Y	Y	Y	Y	Y	Y	Y
12. Were the outcome assessors blinded to the exposure status of participants?	NA	NA	NA	NA	NA	NA	NA	NA	NA	NA
13. Was loss to follow-up after baseline 20% or less?	NA	N	NA	N	N	Y	NA	NA	NA	NA
14. Were key potential confounding variables measured and adjusted statistically for their impact on the relationship between exposure(s) and outcome(s)?	Y	Y	N	Y	Y	Y	Y	Y	Y	Y

*NA* non- available

### Overall effect

We performed a qualitative analysis (Table 3) by combining different frailty measurements and found that the association of frailty risk with TSH levels demonstrated inconsistency among all studies. Moreover, among eight applicable studies, the association of frailty risk with FT4 levels was absent in four studies [21, 24–26] or positive in three studies [20, 22, 23], whereas that of frailty risk with FT3 levels was negative in five [21–24, 26] of six applicable studies.

**Table 3:** Qualitative analysis of the 10 included studies

	**TSH vs frailty**	**FT4 vs frailty**	**FT3 vs frailty**	**Subclinical hyperthyroidism vs frailty**
Positive association	Veronese et al Liu et al	Yeap et al Pasqualetti et al Arosio et al	Nil	Virgini et al
Negative association	Arosio et al Bhalla et al	Nil	Bertoli et al Pasqualetti et al Arosio et al Xiu et al Liu et al	Nil
U-shaped association	Bano et al	Bano et al	Nil	Nil
No association	Yeap et al Pasqualetti et al Xiu et al	Bertoli et al Xiu et al Bhalla et al Liu et al	Bhalla et al	Yeap et al

*TSH* thyroid stimulating hormone, *FT4* free thyroxine, *FT3* free triiodothyronine, *vs* versus

**Fig. 2:**
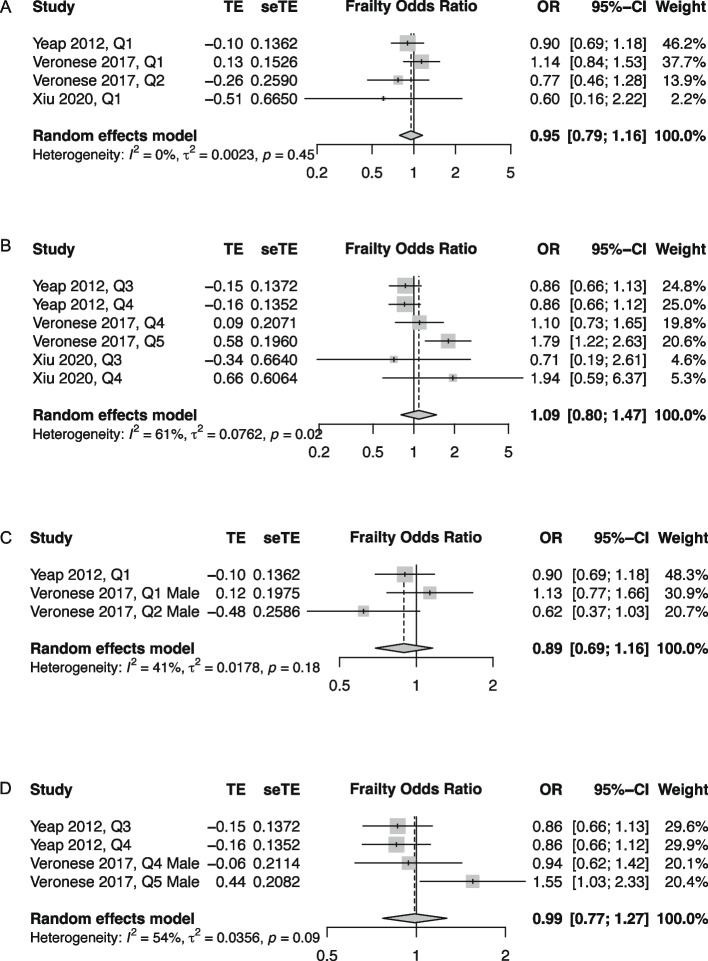
Forest plots of pooled odds ratio for frailty. Footnote: **A** Low and **B** high TSH exposure categories vs. intermediate TSH exposure categories overall. **C** Low and **D** high TSH exposure categories vs. intermediate TSH exposure categories overall in male participants

When integrating data, only the association of TSH (categorical) and frailty phenotype (categorical) could be further analyzed, thus three studies [14, 20, 24] were included in meta-analysis. The data of male participants could be extracted from two studies [14, 20]; however, those of female participants could not. We then integrated the variable cutoffs for TSH exposure among the included studies, divided them into low, intermediate, and high groups (Supplementary Table 2). Comparing the low TSH groups with the intermediate TSH groups and noted that the pooled ORs for frailty were 0.95 (95% CI = 0.79–1.16, *I*^2^ = 0%) in all participants (Fig. 2A) and 0.89 (95% CI = 0.69–1.16, *I*^2^ = 41%) in the male subgroup (Fig. 2C). When high TSH groups were compared with the intermediate TSH groups, the pooled ORs for frailty was 1.09 (95% CI = 0.80–1.47, *I*^2^ = 54%) in all participants (Fig. 2B) and 0.99 (95% CI = 0.77–1.27, *I*^2^ = 54%) in the male subgroup (Fig. 2D).

Given the variations on the cutoffs for TSH exposure among the included studies, no significant association was noted between TSH levels and frailty risk in the pooled results).

### Dose–response meta-analyses

**Fig. 3:**
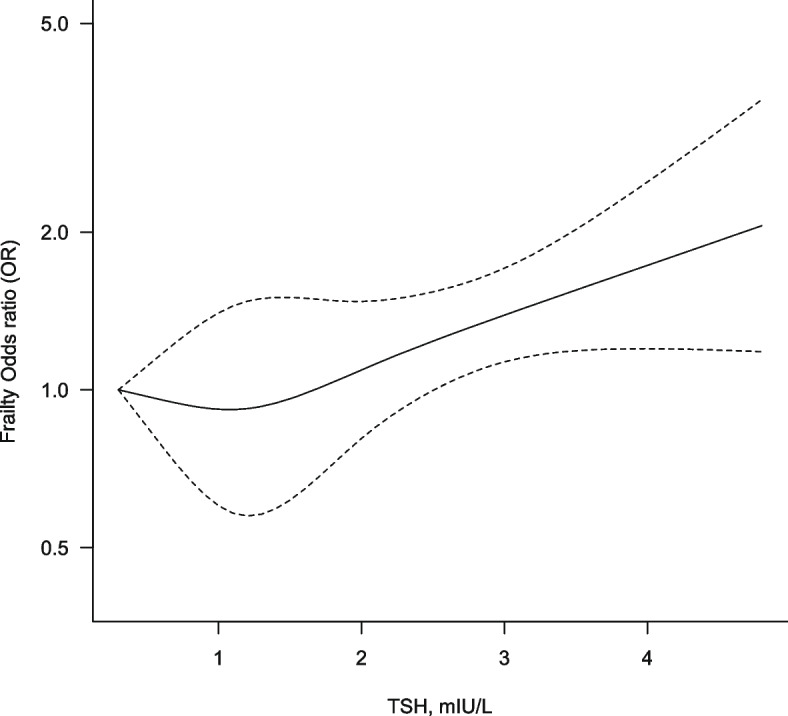
Dose–response association between TSH levels (mIU/L) and frailty risk. Footnote: Restricted cubic splines of random-effects model were used. The solid line represents the estimated odds ratio, and the dashed lines represent 95% confidence interval. TSH, thyroid stimulating hormone

In our dose–response meta-analyses, we noted a significant nonlinear relationship between TSH level and frailty risk (*p* = 0.0071; Fig. 3). An increase of TSH from 0.3mlU/L to 2.7 mlU/L was associated with a significant increase in frailty risk [ORs = 1.30 (95% CIs = 1.06–1.59)]. Moreover, an increase to 4.8 mlU/L from 2.7 mlU/L almost doubled frailty risk [ORs = 2.06 (95% CIs = 1.18–3.57); Table 4]. In addition, TSH levels of 0.6–1.5 mIU/L were not significantly associated with pooled ORs for a frailty of < 1, although the observed ORs were less than 1.

**Table 4:** Predicted ORs (95% CIs) for frailty for different TSH levels

TSH (mIU/L)	OR	95% CI
0.3	1.00	1.00–1.00
0.6	0.96	0.78–1.17
0.9	0.92	0.63–1.35
1.2	0.92	0.57–1.48
1.5	0.96	0.62–1.50
1.8	1.03	0.72–1.48
2.1	1.12	0.85–1.48
2.4	1.21	0.96–1.52
2.7	1.30	1.06–1.59
3.0	1.39	1.13–1.71
3.3	1.48	1.17–1.88
3.6	1.58	1.19–2.10
3.9	1.69	1.20–2.39
4.2	1.80	1.20–2.72
4.5	1.93	1.19–3.12
4.8	2.06	1.18–3.57

*TSH* thyroid stimulating hormone, *OR* odds ratio, *CI* confidence interval

## Discussion

### Summary of evidence

To the best of our knowledge, this is the first meta-analysis on the association between thyroid hormones and frailty in older adults without overt thyroid dysfunction. Qualitative synthesis on the association of frailty with TSH, FT3, FT4, or subclinical hyperthyroidism have reported inconsistent results. Considering the possible J-shaped association, we set the group with intermediate TSH levels as the reference group for our meta-analysis. When we compared the low or high TSH exposure group with the intermediate TSH exposure group, the pooled OR for frailty was nonsignificant. However, the dose–response meta-analyses revealed a significant nonlinear, J-shaped association between TSH levels and frailty. TSH levels within the upper half (2.7–4.8mIU/L) of reference range was noted to significantly increase frailty risk; by contrast, those in the lower half (0.6–1.5 mIU/L) had a lower frailty risk, though nonsignificantly so.

As outlined in the introduction, frailty is a multi-faceted concept with different operational definitions. While the frailty phenotype, characterized by five clinical domains, offers a readily applicable assessment tool; the frailty index, based on a comprehensive accumulation of deficits, provides a more nuanced and sensitive measure of frailty severity. This variability in frailty assessment across studies limits direct comparisons and potentially contributes to the inconsistent findings in the literature. Future research could benefit from developing standardized age-specific cut-off points for the frailty index to facilitate comparisons across studies.

Given that the role of the thyroid hormones in frailty development was not fully established, it remains unclear how overt hyperthyroidism or hypothyroidism affects frailty. Patients with hyperthyroidism demonstrate an increase in muscle protein turnover [27]. Muscle cross-sectional area is smaller in older patients with subclinical hypothyroidism than in age-matched controls with euthyroidism. In the controls with euthyroidism, treatment led to improved muscle strength [28], which possibly explains the J-shaped association observed between thyroid hormones and frailty. The possible mechanism underlying the role of thyroid hormones in frailty development may involve aging and skeletal muscle. Decreases in thyroid hormone production are part of the aging process, during which myogenesis decreases and skeletal muscle metabolism is modulated [6]. Muscle fiber loss with weakening of the remaining neuromuscular junction transmission fibers and instability was also noted [29].

The strength of our study lies in its design: we performed this dose–response meta-analysis to transform the descriptive results into precise quantitative data. Szleif et al. [7] reported a U-shaped association of TSH levels with sarcopenia and low muscle strength, whereas Rong et al. [30] demonstrated a J-shaped relationship between TSH and type 2 diabetes mellitus (T2DM) but an inverted-J-shaped relationships between FT3 and FT4 levels and T2DM. If we assume that this relationship is linear, then frailty risk might not be associated with high or low TSH exposure categories. Therefore, future studies should consider the association between thyroid hormone and frailty to be nonlinear. Future studies should also consider showing subclinical hypothyroidism or hyperthyroidism in a continuous data of FT4 and TSH to extend our J-shaped finding more than reference range (0.3–4.8mIU/L).

### Limitations

First, the studies’ designs of included article were inconsistent with each other. Some studies defined the thyroid hormone as the independent variable and then analyzed the thyroid hormone–frailty correlation or the related ORs. However, other studies divided the participants into frail and nonfrail (prefrail and robust) groups and then measured their blood thyroid hormone levels. Second, the measurement of outcomes was inconsistent between studies: both continuous and categorical thyroid hormone data were used in their analyses. Different cutoff points used for data grouping impeded data integration. In the aspect of frailty, we tried to find a formula to transform continuous data of the frailty index into categorical data. However, the frailty index showed different cut-off points considering different ages [31]. Both the aforementioned limitations resulted in only three studies being included in the meta-analysis. Third, men were overrepresented in the samples of most studies. Underrepresented female participants may cause restriction of future clinical application. Fourth, TSH reference ranges can vary across populations due to factors such as age, ethnicity, and iodine intake. These variations could lead to heterogeneity in the results and potentially attenuate the observed associations between TSH levels and frailty. Fifth, our meta-analysis only included outcome of the frailty phenotype, however its component may overlap with those manifestations from thyroid disorders.

## Conclusion

In the current dose–response meta-analysis, TSH levels and frailty had a significant nonlinear, J-shaped relationship. TSH levels within the range of 2.7–4.8mIU/L were associated with a higher risk of frailty, whereas those within the range of 0.6–1.5 mIU/L showed a trend towards a lower risk of frailty, though this association was not statistically significant. Future studies should also consider showing subclinical hypothyroidism or hyperthyroidism in a continuous data of FT4 and TSH to extend our J-shaped finding more than reference range (0.3–4.8mIU/L).

## Supplementary Information

Supplementary Material 1.

## Data Availability

The datasets used and/or analysed during the current study available from the corresponding author on reasonable request.
