# Supplementary material for: Thyroid hormones and frailty in older adults: systematic review and dose–response meta-analysis
Source: BMC Geriatr. 2025 Feb 17;25:104. doi: 10.1186/s12877-025-05748-5 (PMC11834251; doi:10.1186/s12877-025-05748-5)
Supplement: Supplementary file 1 — Supplementary Material 1. [file 12877_2025_5748_MOESM1_ESM.docx]

**Supplementary Table 1** Search strategy and keywords.

| **Database (Inception date)** | **Searching Strategy** | | | | | | | |
| --- | --- | --- | --- | --- | --- | --- | --- | --- |
| PubMed  (2021/02/02) | **Concepts** | | **Search terms** | | | | | **Results** |
|  | Thyroid  hormone | | #1 | | | "thyroid hormone " | | 117,745 |
|  |  |  | #2 | | | "thyro*" | | 287,942 |
|  |  |  | #3 | | | "TSH" | | 33,579 |
|  |  |  | #4 | | | "thyroxine" | | 61,505 |
|  |  |  | #5 | | | "T4" | | 59,696 |
|  |  |  | #6 | | | "T3" | | 62,625 |
|  |  |  | #7 | | | #1 OR #2 OR #3 OR #4 OR #5 OR #6 | | 373.258 |
|  | frail | | #8 | | | "frail" | | 34,329 |
|  |  |  | #9 | | | "frailty" | | 21,119 |
|  |  |  | #10 | | | #8 OR #9 | | 34,468 |
|  |  | | #11 | | | #7 AND #10 | | 182 |
| EMBASE  PICO Search  (2021/02/02) | **Concepts** | | **Search terms** | | | | | **results** |
|  | Population | | #1 | | | 'adult'/exp | | 143 |
|  | Intervention | |  |  |  | 'thyroid hormone'/exp OR 'thyrotropin'/exp OR 'thyroxine'/exp OR 'liothyronine'/exp | |  |
|  | Comparison | |  |  |  | No restriction | |  |
|  | Outcome | |  |  |  | 'frailty'/exp | |  |
| EBSCOhost  CINAHL Plus with Full Text  (2021/02/02) | **Concepts** | | **Search terms** | | | | | **Results** |
|  | Thyroid  hormone | | #1 | | | thyroid hormone OR thyrotropin OR thyroxine OR T3 OR T4 OR TSH | | 15,798 |
|  | frail | | #2 | | | frailty OR frail | | 18,936 |
|  |  | | #3 | | | #1 AND #2 | | 33 |
| Cochrane  (2021/02/02) | | **Concepts** | | **Search terms** | | | **results** | |
|  |  | Thyroid  hormone | | #1 | Title abstract keyword: " (thyroid hormone) OR (thyro*) OR (TSH) OR (thyroxine) OR (T4) OR (T3) " | | 21061 | |
|  |  | frail | | #2 | Title abstract keyword: " (frail) OR (frailty) " | | 4565 | |
|  |  |  | | #3 | #1 AND #2 | | 45 | |
| Web of Science  (2021/02/02) | | **Concepts** | | **Search terms** | | | **Results** | |
|  |  | Thyroid  hormone | | #1 | (thyroid hormone or TSH or T4 or T3 or thyrotropin or thyroxine).mp. [mp=title, abstract, original title, name of substance word, subject heading word, floating sub-heading word, keyword heading word, organism supplementary concept word, protocol supplementary concept word, rare disease supplementary concept word, unique identifier, synonyms] | | 162033 | |
|  |  | frail | | #2 | (frailty or frail).mp. [mp=title, abstract, original title, name of substance word, subject heading word, floating sub-heading word, keyword heading word, organism supplementary concept word, protocol supplementary concept word, rare disease supplementary concept word, unique identifier, synonyms] | | 33245 | |
|  |  |  | | #3 | #1 AND #2 | | 97 | |

Supplementary table 2. Extracted data of included studies

| **Author** | **Number of participants** | | | **Frailty cases** | **TSH category (mIU/L) ^a^** | | | **Assigned TSH^b^** | **aOR (95% CI)** | **Adjustment Confounders** |
| --- | --- | --- | --- | --- | --- | --- | --- | --- | --- | --- |
| Yeap | 3943 men | | 973 | 147 | Q1 | Low | 0.4^c^–1.4 | 0.9 | 1.00 | Age, BMI, smoke status, diabetes, social support, impairment of seeing or hearing, testosterone and Insulin–like growth factor–I level |
|  |  |  | 968 | 158 | Q2 | Intermediate | 1.41–1.98 | 1.695 | 1.11 (0.85–1.45) |  |
|  |  |  | 967 | 145 | Q3 | High | 1.99–2.8 | 2.395 | 0.96 (0.73–1.25) |  |
|  |  |  | 967 | 152 | Q4 | High | 2.81–4.0^c^ | 3.405 | 0.95 (0.73–1.24) |  |
| Veronese | 2205 | 882 men | 176 | 10 | Q1 | Low | 0.3^c^–0.7 | 0.5 | 1.13 (0.77–1.67) | Age, BMI, smoke status, alcohol drinker, education, monthly income, ADL, geriatric depression, MMSE scores, Charlson comorbidity score, eGFR, number of drugs |
|  |  |  | 177 | 8 | Q2 | Low | 0.7–1.0 | 0.85 | 0.62 (0.41–1.13) |  |
|  |  |  | 176 | 8 | Q3 | Intermediate | 1.0–1.3 | 1.15 | 1.00 |  |
|  |  |  | 177 | 10 | Q4 | High | 1.3–2.0 | 1.65 | 0.94 (0.62–1.42) |  |
|  |  |  | 176 | 13 | Q5 | High | 2.0–4.2^c^ | 3.1 | 1.55 (1.03–2.33) |  |
|  |  | 1323 women | 264 | 28 | Q1 | Low | 0.3^c^–0.8 | 0.55 | 1.14 (0.91–1.42) |  |
|  |  |  | 265 | 23 | Q2 | Low | 0.8–1.1 | 0.95 | 0.89 (0.70–1.13) |  |
|  |  |  | 265 | 20 | Q3 | Intermediate | 1.1–1.5 | 1.3 | 1.00 |  |
|  |  |  | 265 | 23 | Q4 | High | 1.5–2.5 | 2 | 1.22 (0.96-1.54) |  |
|  |  |  | 264 | 40 | Q5 | High | 2.5–4.2^c^ | 3.35 | 1.97 (1.59-2.45) |  |
| Xiu | 240 | | 61 | 5 | Q1 | Low | 0.55^c^-1.15 | 0.85 | 1.00 | Age, sex, FT3, eGFR, and 25(OH) D3 |
|  |  |  | 59 | 8 | Q2 | Intermediate | 1.16-1.63 | 1.395 | 1.66 (0.45–6.10) |  |
|  |  |  | 59 | 8 | Q3 | High | 1.64-2.36 | 2 | 1.18 (0.32–4.32) |  |
|  |  |  | 61 | 15 | Q4 | High | 2.37-4.78^c^ | 3.575 | 3.22 (0.98–10.56) |  |

^a^Pooling the ORs of frailty among different cutoff values of TSH for meta-analysis in comparison with low/high vs. intermediate level

^b^Midpoint of TSH assigned for dose–response meta-analysis

^c^Reference value of TSH from each study
